# Protective Community Norms and Mental Health Risks for Severe Physical Abuse: Lessons From a Nationally Representative Study of Ghana

**DOI:** 10.1177/08862605231156418

**Published:** 2023-02-26

**Authors:** Clifton R. Emery, Alhassan Abdullah, Lucy P. Jordan

**Affiliations:** 1 The University of Hong Kong, Pok Fu Lam, Hong Kong; 2 Flinders University, Adelaide, SA, Australia

**Keywords:** Abiriwatia, collective childcare, borderline personality disorder, physical abuse, community norms, social norms, Ghana

## Abstract

Although it has become axiomatic to quote an African proverb in discussions of child well-being, attempts to draw concrete and positive lessons from how African communities respond to and mitigate child maltreatment are comparatively few. This study tested the hypothesis that the collective value of Abiriwatia in Ghana, which supports legitimate norms of community obligations to care for children, could be protective against physical abuse. It also examined the claim that knowledge of the familial situation of community members, generated through Abiriwatia, may help them to act to mitigate the risk of caregiver’s borderline personality disorder (BPD) features. We obtained a nationally representative sample of 1,100 female caregivers from 22 Ghanaian settlements and tested the hypotheses using multilevel models. Controlling for community-level physical abuse, living in a community with high levels of Abiriwatia childcare and community authority values is associated with lower levels of very severe physical abuse, and Abiriwatia childcare may mitigate risk from the caregiver’s BPD features. Within Ghana, encouraging positive and protective aspects of traditional Ghanaian values and working to reinstate respect for these values may have positive outcomes for children. Interventions to reduce child maltreatment should be developed with reference to Abiriwatia childcare values.

## Introduction

Physical abuse affects 22.6% of children worldwide (Stoltenborgh et al., 2013) and 43% of children in Ghana, which is also the median prevalence for Africa (Akmatov, 2011). Although it has become axiomatic to quote an African proverb in discussions of child well-being (Verhoef, 2005), attempts to draw concrete and positive lessons from how African communities respond to and mitigate child maltreatment are comparatively few (but see Abdullah, Frederico, et al., 2020; Verhoef, 2005, for exceptions). It may well be the case that “it takes a village to raise a child” (Verhoef, 2005), but if the claim is taken seriously then it behooves researchers to examine precisely *how* African villages successfully raise children and apply those lessons to better protect children everywhere. This study begins to address the research gap by examining the Ghanaian community value of Abiriwatia at the community level in a nationally representative study of 1,100 Ghanaian female caregivers in 22 Ghanaian settlements. Abiriwatia is a lineage-based Ghanaian value that comprises norms mandating collective childcare; recent research shows it is negatively associated with severe neglect (Abdullah et al., 2023, forthcoming). At the individual level, the study examines whether a known parental mental health risk factor in Western countries (Hiraoka et al., 2016), borderline personality features, similarly puts children at risk for physical abuse in Ghana. Borderline personality disorder (BPD) is often considered as one of the most severe personality disorders and is characterized by enduring patterns of impulsivity, identity disturbance, labile affect, and emotionally intense, tumultuous interpersonal relationships (American Psychiatric Association [APA], 2013; Hiraoka et al., 2016). Hence, the study contributes to a deeper understanding of severe physical abuse by combining African community characteristics and a known (Western) risk factor for severe physical abuse in a single model using Ghanaian data.

Child maltreatment results in millions of lost Daily-Adjusted Life Years in sub-Saharan Africa alone (Fang et al., 2017). Physical abuse puts children at higher risk for suicide (Mironova et al., 2011), depression and anxiety (Lindert et al., 2014), aggression (Keene & Epps, 2016), substance abuse (Halpern et al., 2018), posttraumatic stress disorder and other forms of psychopathology (Adams et al., 2018), behavior problems (Emery, Trung, et al., 2015), school dropout and other academic problems (Gubbels et al., 2019), and ultimately early mortality (Grummitt et al., 2021). In the United States alone, 1,374 children under 5 years of age die from maltreatment each year; two-thirds of these deaths are attributable to physical abuse (Klevens & Leeb, 2010). Accurate estimates outside of industrialized countries are difficult to obtain.

About 4 out of every 10 children in Ghana experience abuse (Akmatov, 2011). Boys living in large rural households and who do not live with their biological parents are at particular risk for physical abuse in Ghana (Adjorlolo et al., 2017). Children with disabilities may be at a substantially increased risk for physical abuse (Kassah et al., 2012). Children who are physically abused in Ghana are at increased risk for depression, low self-efficacy, and low life satisfaction later in life (Adjorlolo et al., 2017). Even when severe cases of abuse are detected via X-rays, official reporting in Ghana can be hindered by fear of retaliation (Antwi et al., 2019) or to “save face” (Abdullah, Emery, et al., 2020; Abdullah, Manful, et al., 2020). As in many developing countries, child protection services in Ghana are under-resourced (Krueger et al., 2014), and protection principles are mainly borrowed from Western concepts without consideration of important local cultural characteristics (Manful, Abdullah, Cudjoe, 2020; Manful, Cudjoe, Abdullah, 2020). Unsurprisingly, these systems prove to be difficult and costly to implement and are only able to touch the tip of the child maltreatment iceberg (Krueger et al., 2014). Moreover, the formal system fails to leverage existing community practices in Ghana that have evolved to prevent and mitigate the most severe forms of maltreatment (Abdullah, 2022; Abdullah, Frederico, et al., 2020).

### Abiriwatia

Abiriwatia is a Ghanaian communal value centered around people believed to be descendants of a common ancestor, who is putative (Nukunya, 2003). Abiriwatia integrates communities vertically across generations (Abdullah et al., 2023). The normative dimensions of Abiriwatia include support for (a) the authority of family and community leaders, (b) community obligations to care for children, and (c) abiding by taboos and lineage succession rules (Abdullah et al., forthcoming; Nukunya, 2003).

New research has found a negative relationship between female caregivers’ endorsed adherence to Abiriwatia and frequency of severe neglect (Abdullah et al., 2023), but we know of no research on the relationship between Abiriwatia and physical abuse. Moreover, Abdullah et al. (2023) argue for an individual-level Abiriwatia mechanism; mothers have internalized Abiriwatia norms that (negatively) sanction neglect, resulting in less neglect. Our argument and approach to Abiriwatia are somewhat different. At an individual level internalized norms place limits on sanctioned behaviors, forcing actors to excuse or condone deviance when they violate those norms (Matza, 1964/2017). Durkheim (1982) argues that sanctions for deliberate violation of legitimate norms, at least start internally within the actor concerned, and manifest in terms of guilt and shame. But efforts to ensure conformity to institutionalized norms, through positive and negative situational sanctions, can reinforce commitments even among those who were not committed to the norm within the social order. Research in behavioral psychology suggests that conformity to norms can also reinforce commitment. Cialdini (2006) provides evidence that conformity (consistency) may increase commitment (study participants were induced to cut energy costs by the incentive of being honored publicly in the newspaper, but adopted more environmentalist beliefs when the honor was *not* provided). In cross-sectional studies this reverse channel causes bidirectional causality bias in estimates of relationships (Johnston & DiNardo, 1997). Coefficients in linear models will capture both the effect of Abiriwatia norms on neglect and the effect of neglect (or non-neglect) on Abiriwatia norms.

Abdullah et al. (2023) partially dealt with the bidirectionality bias problem by only looking at female caregivers who reported at least some neglect, excluding those with no neglect. Our approach is different. Because Abiriwatia is a collectivist value, we measure it at the community level (settlement average) and model its relationship with individual female caregiver level very severe physical abuse using multilevel models. If community-level physical abuse is controlled for, this approach can control for bidirectional bias at the community level while still estimating the relationship between community-level Abiriwatia and individual-level physical abuse by female caregivers. The theoretical logic follows theories of persuasion and influence^1^ (Cialdini, 2006; Gould, 1987; Luhmann, 1976; Parsons, 1937). Influence is, by definition, contagious. By this logic, communities high in the collective responsibility for the childcare dimension of Abiriwatia should affect female caregivers’ decision-making, whether or not they share those norms. Severe physical abuse would be considered more deviant in communities high in Abiriwatia than those low in it, with a concomitant loss of local respect for the perpetrator. This leads to the first hypothesis.

Hypothesis 1: Community-level Abiriwatia childcare norms will be negatively associated with individual-level physical abuse severity by female caregivers.

### Borderline Features

Although there is a long history of research on the relationship between BPD and severe and life-threatening intimate partner violence (IPV) (Holtzworth-Munroe & Meehan, 2004; Holtzworth-Munroe & Stuart, 1994), research on the relationship between BPD features and physical abuse of children remains limited (Hiraoka et al., 2016). Three previous studies examined mothers who were substantiated for child maltreatment. In all, substantiation was associated with elevated BPD features (Laulik et al., 2016; Perepletchikova et al., 2012). Hiraoka et al. (2016) found a strong relationship between physical abuse risk (using the Child Abuse Potential Inventory) and BPD features in a general population study. However, very little research has examined BPD features and reported physical abuse in a general population study, and we know of no such research in Ghana. This is a concern because abuse cases in the formal child protection system represent only a small proportion of the total (Gilbert et al., 2009). Hence, the overall impact of BPD features on physical abuse in the population is unclear.

Theoretically, BPD features may increase risk of physical abuse because of associated difficulties with emotional regulation (Scott et al., 2014). Specifically, BPD features are associated with rage in the face of perceived rejection or abandonment (Berenson et al., 2011). The rejection sensitivity model (Downey & Feldman, 1996) argues that BPD is associated with both intense, uncontrolled responses to rejection and processing biases that render such individuals more likely to perceive rejection even in benign situations, creating risk of a self-fulfilling relationship prophecy (Berenson et al., 2011; London et al., 2007). A parent’s high levels of BPD features may put children at high risk for severe physical abuse when the parent has both a high level of rejection sensitivity and sees the child as a “friend” or otherwise as an important source of emotional support. Hence, we hypothesize that female caregivers’ BPD features will be positively associated with physical abuse of the child.

Hypothesis 2: Female caregivers’ BPD features will be positively associated with physical abuse severity by female caregivers.

Communities that are high in Abiriwatia childcare values may be more consistently responsive to child maltreatment, mitigating child maltreatment. When such values lead community members to conclude “my neighbor’s child is my own,” they may be more likely to become familiar with the family circumstances and characteristics of the caregivers who perpetrate abuse and the children who are physically abused (including a caregiver’s BPD features). Familiarity with the situations that provide the caregiver with an emotional cue to perpetrate abuse may help community members to monitor more closely or even act preemptively. Findings from a Ghanaian study show that in communities that are high in Abiriwatia values, members are more likely to undertake social control practices to prevent and remedy child neglect situations (Abdullah et al., 2023). Emery, Eremina, et al.’s (2015) study in Seoul revealed that protective informal social practices, in the face of physical abuse, can mitigate severe abuse injuries. Informal social control practices include surveillance practices and actual actions that are undertaken by community members to remedy violence and maltreatments, including physical abuse (Emery, Trung, et al., 2015). The strength of Abiriwatia values moderated the relationship between acts of informal social control and the frequency of child neglect. For these reasons we hypothesize that community-level Abiriwatia childcare values will moderate the relationship between individual-level BPD features and physical abuse of the child.

Hypothesis 3: Community-level protective Abiriwatia childcare values will have a negative interaction with female caregivers’ BPD features in predicting physical abuse severity.

### The Current Study

The current study contributes to the literature on physical child abuse as it is the first study to examine physical abuse in relation to Abiriwatia, and one of the very few studies that examine BPD features in relation to physical abuse. In examining the protective effects of Ghanaian culture regarding physical abuse, this study departs from the pattern of characterizing the severity of abuse in African countries, and instead shifts focus to positive lessons that can be learned in the West African context. Moreover, the study produces prevalence estimates for Ghana as it drew on a rigorously conducted random stratified multistage cluster sample representative of both rural and urban Ghana. Clustering by settlement allowed the study to control for multiple potential confounds at the community levels, including community-level average education, household income, age, years married/cohabiting, years resident, social trust, and collective efficacy, and whether the community was rural or urban, which are known to be related to crime (Sampson et al., 1997). As discussed above, the study controlled for community-level physical abuse severity as a means of mitigating bidirectional causality bias in the relationship between community-level Abiriwatia and individual-level physical abuse severity. At the individual level the models controlled for the focal child’s age, sex, the number of children in the family, the number of people living in the household, whether the house was an apartment or a compound house, and the female caregiver’s age. The models also controlled for the caregiver’s desire to keep neglect secret, which we implement as a measure of social desirability. Because traditional community thresholds for consideration of what constitutes problematic violence against children may tolerate more severe violence than the law allows, we use very severe physical abuse as an outcome measure.

## Methods

### Participants and Procedure

The data are a stratified four-stage random probability proportional to size (PPS) cluster sample of 1,100 female caregivers in Ghana. The sample was stratified 41% urban and 59% rural. Rural settlements were oversampled (Ghana is 57.3% urban) because of the study’s focus on traditional Ghanaian community values and practices, which are considered to be strong in rural areas (Nukunya, 2003). Using Ghanaian census data, seven districts (three urban, four rural) were randomly selected using a PPS approach. Again, using PPS, 22 settlements (at least three per district) were randomly selected within the seven districts. In each settlement a map-based, next-door-neighbor cluster, area-based sampling approach was used to select 50 female caregivers. In each settlement, five start points were selected using random draws from a uniform distribution (two draws per start point to obtain random coordinates). Interviewers were trained to locate the residences closest to the start points and interview the 10 female caregivers living closest to each start point. Only female caregivers were sampled in this study since women are the primary caregivers of children in traditional African communities (Nukunya, 2003). And it is expected that they are more likely to abuse children due to the frequent contact with the children. Interviewer training included informed consent, sensitivity to child maltreatment and family violence, role-playing, and a written exam and certification interview. Institutional Review Board (IRB) approval of the written informed consent procedure was obtained from the University of Hong Kong. Masks were distributed to study participants. The study had a 95% response rate. A local academic translated the questionnaire into Twi language. The questionnaire was then translated back to check for accuracy. A sample size of 1,100 female caregivers allows for a margin of error of ±3% in prevalence estimates for Ghana.

### Measures

#### Physical abuse severity

Severity of physical abuse of the child was measured using items from the Conflict Tactics Scale 2 (CTS2; Straus et al., 1998). Fourteen items asked whether the female caregiver had done the following to the focal child in the past year or ever: (1) shaken, (2) hit on the bottom with a hard object, (3) punched or kicked, (4) choked, (5) beaten up, (6) deliberately burned or scalded, (7) hit some other part of the body with a hard object, (8) slapped or caned him/her on the hand, arm, or leg, (9) pinched, (10) used or threatened to use a knife or gun, (11) threw or knocked him/her down, (12) slapped on the face, head, or ears, (13) gave a punishment that caused cuts/bruises, (14) gave a punishment that resulted in a need to consult a doctor (MD). Possible responses were once in the past year, twice in the past year, 3 to 5 times in the past year, 6 to 10 times in the past year, 11 to 20 times in the past year, more than 20 times in the past year, not in the past year but did happen before, and never happened. Cronbach’s α for the first 12 items was .72. Very severe abuse was limited to the following five items: (4) choked, (5) beaten up, (6) deliberately burned or scalded, (10) used or threatened to use a knife or gun, and (11) threw or knocked him/her down. The last two (injury) items were used to create a scale from these five items in the following manner.

Emery, Thapa, et al. (2015) note that although CTS items capture frequencies, summing frequencies across different levels of violence compares apples to oranges when it comes to injury. To remedy this problem, they created an IPV scale composed of CTS item frequencies weighted by the item’s associated log odds of producing an injury. This weighted approach ensures an apples-to-apples comparison because each reported act of physical violence is weighted by its log odds of producing injury. Very injurious acts of violence are inflated on the scale, while less injurious acts have their influence reduced. Although we note the advantages of this approach for creating a physical abuse severity scale, we also note a limitation. The original CTS items are frequencies (counts) of violence in the past year. They are appropriately modeled with a Poisson model rather than a regression model (Kennedy, 1998). However, the weighting approach creates decimals which prevent the use of a Poisson model. To remedy this problem, we first followed Emery, Thapa, et al., (2015) in creating a physical abuse severity scale by weighting each item’s frequency (count) by its associated log odds (from bivariate logistic regressions) of producing injury, and then summing the weighted frequencies. To appropriately analyze the scale’s weighted counts using a Poisson model, we multiplied the physical abuse severity scale by 10 and rounded to the nearest whole number. The resulting scale ranged from 0 to 84 and had a mean of 1.86 and a standard deviation of 7.22.

#### Abiriwatia

Following Abdullah et al. (forthcoming), Abiriwatia was measured using the following 11 items: (1) this community has a collectively agreed on authority when it comes to family affairs, (2) in this community people tend to listen to that authority figure, (3) the authority figure makes decisions that are binding for members of the family, (4) this authority figure greets me whenever she/he sees me, (5) the upbringing of children is the collective responsibility of everyone in the community, (6) my neighbor’s child is my own child, (7) children are products of communities, (8) caring for a child in the neighborhood is part of what it means to be a member of this community, (9) I owe allegiance to my lineage, (10) commitment to my lineage (forebears) is a value that still informs my decisions and daily life, and (11) I believe all members of my family are descendants of a common ancestor. A four point Likert scale (*strongly disagree* to *strongly agree*) was used to capture agreement. Cronbach’s α was .87. The Abiriwatia scale was divided into three subscales, each of which was summed across items. The respect for forebears and lineage subscale was composed of the following items: (9) I owe allegiance to my lineage, (10) commitment to lineage (forebear) is a value that still informs decisions and daily life, and (11) I believe that all members of my family are descendants of a common ancestor, and had a Cronbach’s alpha of α = .77. The community-based authority subscale was composed of the following items: (1) this community has a collectively agreed on authority when it comes to family affairs, (2) in this community people tend to listen to that authority figure, (3) the authority figure makes decisions that are binding for members of the family, (4), this authority figure greets me whenever she/he sees me, and had a Cronbach’s alpha of α = .97. The community childcare responsibility subscale was composed of the following items: (5) the upbringing of children is the collective responsibility of everyone in the community, (6) my neighbor’s child is my own child, (7) children are products of communities, (8) caring for a child in the neighborhood is part of what it means to be a member of this community, and had a Cronbach’s alpha of α = .87. Settlement-level averages were then calculated from individual scores on the scales.

#### Borderline personality features of female caregiver

Following Hiraoka et al. (2016), we measured borderline personality features using the 10-item yes/no self-report McLean Screening Instrument for Borderline Personality Disorder (MSI-BPD) (Zanarini et al., 2003). The following were the items: (1) have any of your closest relationships been troubled by a lot of arguments or repeated breakups, (2) have you deliberately hurt yourself physically (e.g., punched, cut, burned yourself), have you made a suicide attempt, (3) have you had at least two other problems with impulsivity (e.g., binge eating and spending sprees, drinking too much and verbal outbursts), (4) have you been extremely moody, (5) have you felt very angry a lot of the time? How often have you acted in an angry or sarcastic manner, (6) have you often been distrustful of other people, (7) have you frequently felt unreal or as if things around you were unreal, (8) have you felt chronically empty, (9) have you often felt that you had no idea of who you are or that you have no identity, and (10) have you made desperate attempts to avoid feeling abandoned or being abandoned (e.g., repeatedly called someone to reassure yourself that he or she still cared, begged them not to leave you, clung to them physically)? The MSI-BPD has good criterion validity regarding BPD, with 81% correct classification of borderline patients (sensitivity) and 85% correct classification of non-borderline cases (specificity) (Patel et al., 2011; Zanarini et al., 2003). As the items were dichotomous, we used Kuder–Richardson formula 20 to assess internal reliability (α = .70). Items were summed to create a 0 to 10 point scale.

### Control Variables

#### Community-level collective efficacy

Collective efficacy was measured using a modified version of Sampson et al.’s (1997) measure (Emery, Trung, et al., 2015). All items used a four-point Likert scale ranging from “*strongly agree*” to “*strongly disagree*.” Community solidarity was measured with the following items: (1) my neighborhood is a close-knit community, (2) people in this community generally get along with each other, (3) people in this community can be trusted, (4) people around here are willing to help their neighbors, and (5) people in this community share the same values (Cronbach’s α = .89). Community informal social control was measured with the item “You could count on your neighbors to do something about it if”: (1) children were skipping school and hanging out on a street corner, (2) a child was showing disrespect to an adult, (3) there was a fight in front of your house/apartment and someone was being beaten or threatened, (4) children were spray-painting graffiti on a local building, and (5) people are seen stealing through your window (Cronbach’s α = .85). Cohesion and informal social control items were then summed to make separate scales, and settlement-level averages of the scales were calculated.

#### Community-level physical abuse

As discussed above, settlement-level averages of physical abuse severity were calculated and controlled for to mitigate bidirectional causality bias in the models. The measure of physical abuse severity came from the Conflict Tactics Scale 2 (CTS2; Straus et al., 1998).

#### Community-level social trust

A single item captured social trust. “On a scale of 1 to 6 on which a 1 means most people can be trusted and 6 means you can’t be too careful, please answer: Generally speaking, would you say that most people can be trusted or that you can’t be too careful in dealing with people?” (Torpe & Lolle, 2011). This is a forced choice question in which responses 1 to 3 are under the heading “most people can be trusted” and responses 4 to 6 are under the heading “can’t be too careful.” Settlement-level average scores were calculated and included as a control in the models.

#### Neglect secrecy

The participants’ tendency to keep neglect secret was controlled for as a measure of social desirability about reporting child maltreatment. The items were, “If I could not take good enough care of my child, I would try to keep it secret from”: (1) my friends, (2) my family, (3) my neighbors, (4) my co-workers, (5) my boss, and (6) everyone. Possible responses were *strongly agree*, *agree*, *disagree*, and *strongly disagree* (Cronbach’s α = .83).

#### Demographic controls

Community-level demographic controls included settlement-level averages for female caregiver’s age and education (% who completed middle school), median household income, average years resident and years married, and whether the community was rural or urban. At the individual level, models controlled for the focal child’s age and sex, the number of children in the family, the number of people living in the household, whether the housing type was an apartment or compound house, and the female caregiver’s age.

### Analytic Issues

Hypotheses were tested using multilevel Poisson regression models with random effects for each settlement cluster using *Stata 17*. Diagnostics were run on the main effects model. Pregibon’s link test showed significant nonlinearity (hat-squared = .05, *t* = 6.45, *p* < .001). Box-Tidwell transformations were undertaken to identify the chief sources of nonlinearity. Box-Tidwell models suggested borderline personality features, focal child’s age, and neglect secrecy to be the largest contributors to nonlinearity. Lowess smoothing with a bandwidth of 0.4 was used to help identify appropriate function forms for BPD features, the focal child’s age, and neglect secrecy. Smoothed fits for both BPD features and neglect secrecy were both consistent with parabolic (squared) relationships. Hence, both linear and squared terms for both variables were included in the models. However, the smoothed relationship for focal child’s age was unusual, bearing a strong relationship in form to the letter M. This implies focal child’s age to the fourth power significantly predicts the very severe abuse severity outcome. When linear, squared, cubed, and fourth power focal child’s age variables were included in the model, all were highly significant and were retained in the model. The squared term for BPD features was significant when added to the model (*B* = 0.008, *p* < .05). However, when powers of the focal child’s age were added it became marginally significant (*B* = 0.007, *p* = .058). The BPD feature’s squared term was retained in the model however as nonlinearity in one of the two principal independent variables has serious theoretical import. Studentized residuals were calculated to test for outliers. Appropriate effect sizes were Bonferroni corrected for the sample size (*t* = 4.09 for *p* < .05 significance), resulting in 12 outliers. When these 12 observations were removed from the main effects model the BPD feature’s squared term became larger and highly significant (*B* = 0.01, *p* < .01). Coefficients for other predictors of interest did not change in sign or significance. All observations are retained in the final models. The 12 observations are included in the analyses shown, rendering the findings statistically conservative.

## Results

Table 1 reports means (percentages) and standard deviations of the variables in the models for rural and urban areas as well as combined to be representative of Ghana. The combined estimates are re-weighted to represent the correct rural/urban ratio and consequently function as national prevalence estimates. Significance tests shown are *t*/*Z* tests for differences in rural versus urban means.

**Table 1.** Sample Descriptive Statistics.

Variable	Overall Country		Urban		Rural	
	Mean	Standard Deviation	Mean	Standard Deviation	Mean	Standard Deviation
Physical abuse severity	1.59	7.22	0.83***	3.25	2.61	8.99
Child’s age	7.88	4.36	7.49***	4.37	8.40	4.29
Female child	50.3%		49.7%		51.2%	
Number of children	2.60	1.51	2.45***	1.43	2.81	1.54
Caregiver’s age	38.27	9.78	38.03*	8.77	38.60	10.42
Rural	43%					
Borderline personality features	4.70	2.39	4.58***	2.37	4.85	2.41
Social trust	3.87	1.26	3.81**	1.28	3.94	1.25
Child neglect	55.66		38.4%***	0.49	0.79%	
Sustained protective informal social control	5.87	9.14	4.10***	8.20	8.23	9.37
Sustained punitive informal social control	0.03	1.06	0	0	0.06	1.38
Abiriwatia lineage	2.94	0.76	2.65***	0.69	3.31	0.70
Abiriwatia community authority	3.14	0.83	2.79***	0.93	3.60	0.57
Abiriwatia community childcare	3.53	0.48	3.44***	0.48	3.64	0.46
Years resident in community	12.60	11.51	11.37***	8.75	14.24	12.89
Years married	11.59	9.70	11.07***	8.80	12.30	10.23
Community solidarity	2.91	0.65	2.86***	0.66	2.98	0.64
Community informal social control	3.46	0.50	3.35***	0.51	3.60	0.46
Education	49.4%		49.1%		50.1%	
Household income	214.20	304.03	218.19	332.62	208.84	282.97

* *p* < .05 (rural urban difference).

** *p* < .01 (rural urban difference).

*** *p* < .001 (rural urban difference).

The whole country average for physical abuse on the severe abuse severity scale was 1.59 with a standard deviation of 7.22. Rural areas had a significantly higher physical abuse severity average than urban areas. All three types of Abiriwatia values were significantly more strongly endorsed in rural households and sustained protective ISC_CM was more common among rural households than urban households. The borderline feature’s average was significantly higher among rural female caregivers. Neighborhood solidarity and informal social control (collective efficacy) were both significantly higher in rural settlements. Rural households were also significantly higher in social trust. Rural households had significantly more children living in them than urban households, and the focal child was significantly older. Rural respondents had also been married significantly longer and lived in the same settlement for more years on average 50.6% of the focal children in the study were female, and the average age of focal children was 7.9 years old. Households on average had 2.6 children. The average female caregiver was 38.3 years old and had been married or cohabiting for 11.6 years. The household income of these women was, on average, 214 USD per month; 49% of women in the sample had finished middle school but not obtained further education.

Prevalence of physical abuse in Ghana was high. Overall prevalence of any past year physical abuse in Ghana was 71.35%. Physical abuse prevalence in rural Ghana was higher than in urban, but not significantly so. However, other items differed significantly across the rural–urban gap. Past year very severe physical abuse (knocked down, beat up, choked, burned, and used or threatened to use a knife or gun) was significantly more common in rural (23.6%) than in urban (10.7%) areas (*t* = 5.50, *p* < .001). Overall weighted prevalence for past year very severe physical abuse in Ghana was 16.19%. Each of all five very severe physical abuse items was more common in rural rather than in urban areas. Table 2 provides a detailed breakdown of lifetime (not past year) prevalence estimates of different types of physical abuse by urban and rural households, as well as overall lifetime prevalence estimates of each type in Ghana. Children’s lifetime prevalence of being beaten up in Ghana was high (19.6%). Being knocked down was the next most common experience (6.6%). Having caregivers threaten with or use a knife or gun (1.3%) was the third most common form of very severe abuse. Being choked (0.98%) or deliberately burned (0.53%) were comparatively rare.

**Table 2.** Lifetime Prevalence of Types of Abuse by Urban, Rural, and Overall.

Variable	Overall Country Prevalence		Urban		Rural	
	Mean	Standard Deviation	Mean	StandardDeviation	Mean	Standard Deviation
Shaken	0.25	0.43	0.27**	0.44	0.23	0.42
Hit him/her on the bottom with a hard object	0.67	0.47	0.64***	0.48	0.70	0.46
Punched/kicked	0.13	0.37	0.06***	0.23	0.24	0.43
Choked	0.01	0.11	0.004***	0.07	0.02	0.13
Beaten up	0.20	0.41	0.14***	0.35	0.26	0.44
Deliberately burned or scalded	0.01	0.08	0.00***	0.00	0.01	0.10
Hit some other part of the body with a hard object	0.46	0.50	0.41***	0.49	0.53	0.50
Slapped or caned him/her on the hand, arm, or leg	0.73	0.45	0.76***	0.42	0.69	0.47
Pinched	0.21	0.40	0.24***	0.43	0.16	0.38
Used or threatened to use a knife or gun	0.01	0.13	0.002***	0.05	0.03	0.17
Threw or knocked him/her down	0.07	0.26	0.05***	0.22	0.09	0.28
Slapped on the face, head, or ears	0.14	0.36	0.08***	0.27	0.22	0.41
Gave a punishment that caused cuts/bruises	0.04	0.20	0.03***	0.17	0.05	0.22
Gave a punishment that resulted in a need to consult a doctor (MD)	0.01	0.10	0.01	0.09	0.01	0.10

* *p* < .05 (rural urban difference).

** *p* < .01 (rural urban difference).

*** *p* < .001 (rural urban difference).

Table 3 shows the main effects and interaction models. The first column of coefficients in Table 3 shows the main effects model. Both the BPD feature’s linear and squared terms are positive and jointly significant (χ^2^ = 427.8, *df* = 2, *p* < .001), indicating an upward opening parabola that opens steeply upward for higher values of BPD features. Unit increases in very severe physical abuse associated with BPD features accelerate at higher values of BPD features. Living in a community with high levels of Abiriwatia childcare values (*B* = −4.35, *p* = .029) and high levels of Abiriwatia community authority (*B* = −1.91, *p* = .027) is significantly associated with lower levels of very severe abuse. The focal child’s age is strongly associated with very severe physical abuse. The significant fourth power polynomial indicates that age has an M shape with the middle sharp dip in very severe physical abuse occurring around the age of 10. Controlling for the number of children in the household, larger households were associated with lower levels of very severe abuse (*B* = −0.01, *p* = .012). Older female caregivers were associated with lower levels of very severe abuse (*B* = −0.02, *p* < .001). The squared term for secrecy about neglect is significant and positive, indicating an upward opening parabola (because the second derivative is positive this indicates a minimum). Hence, higher levels of secrecy are associated with large, nonlinear increases in very severe abuse. At the community level, higher levels of social trust (*B* = −1.32, *p* = .012), neighborhood informal social control (*B* = −6.73, *p* < .001), and with residents higher, on average, in age (*B* = −0.16, *p* = .048) were associated with lower levels of very severe abuse. Communities with residents who had lived there longer had, on average, higher levels of very severe abuse (*B* = 0.07, *p* = .013).

**Table 3.** Random Effects Poisson Regression Models on Physical Abuse Severity (*N* = 1,019).

Variable	Main Effects		Abiriwatia X Borderline	
	*B*	*SE*	*B*	*SE*
Borderline personality features	0.14***	0.04	−0.54	0.31
Borderline personality features squared	0.01	0.003	0.07***	0.02
Child’s age	2.31***	0.23	2.35***	0.24
Child’s age squared	−0.39***	0.04	−0.40***	0.04
Child’s age cube	0.03***	0.003	0.03***	0.003
Child’s age fourth power	−0.001***	0.00	−0.001***	0.00
Female child	0.22***	0.05	0.21***	0.05
Number of children	0.003	0.02	−0.001	0.02
Caregiver’s age	−0.02***	0.002	−0.02***	0.002
Type of household	−0.06	0.09	−0.07	0.09
Household size	−0.01**	0.004	−0.01*	0.004
Rural	−0.36	0.89	−0.43	0.90
Neglect secrecy	−0.54***	0.14	−0.55***	0.14
Neglect secrecy squared	0.12***	0.02790	0.12***	0.03
*Interactions*				
Abiriwatia community childcare X borderline			0.12	0.08
Abiriwatia community childcare X borderline squared			−0.001***	0.00
*Community level*				
Social trust	−1.32*	0.55	−1.32*	0.55
Caregiver’s age	−0.16*	0.08	−0.17*	0.08
Years resident in community	0.07**	0.03	0.07*	0.03
Abiriwatia lineage	1.28	0.95	1.32	0.95
Abiriwatia community authority	−1.91*	0.86	−1.86*	0.87
Abiriwatia community childcare	−4.35*	1.99	−4.67*	2.04
Years married	0.19*	0.08	0.19*	0.08
Community solidarity	0.07	1.14	−0.04	1.15
Community informal social control	−6.73***	1.87	−6.65***	1.88
Education	−1.54	2.31	−1.44	2.32
Household income	0.001	0.004	0.001	0.004
Physical abuse severity	0.15	0.09	0.16	0.09

* *p* < .05. **p < .01. ***p < .001.

The second column of coefficients in Table 3 shows the model with the test of the hypothesized Abiriwatia X BPD features relationship. In this model that includes the interaction terms, the BPD features squared coefficient has become highly significant (*B* = 0.07, *p* = .001). Also, higher levels of Abiriwatia community authority values have become negatively associated with very severe abuse in this model (*B* = −1.86, *p* = .032). Because the relationship of BPD features with very severe abuse is captured with both a linear and a squared term, testing the interaction requires testing an interaction between community-level Abiriwatia childcare values and each of the two BPD features’ terms. Although the linear interaction term is not significant, the interaction between Abiriwatia childcare values and BPD features squared is negative and highly significant (*B* = −0.001, *p* = .004). Although squared interactions are relatively rare, they can and are sometimes predicted for theoretical reasons (Emery & Yang, 2022). In this case, the negative interaction with the squared term as opposed to the positive coefficient for the squared BPD features’ term suggests that high community-level Abiriwatia values may be associated with an attenuation of high levels of very severe abuse associated with very high levels of BPD features.

## Discussion and Implications

The findings suggest that 16.19%, more than one out of every six children in Ghana, have experienced very severe physical abuse in the past year. Physical abuse remains a severe problem, with nearly three out of every four (71.4%) children experiencing some form of physical abuse in the past year. Based on previous research, hypothesis one predicted a negative relationship between Abiriwatia childcare values at the community level and very severe physical abuse. This hypothesis was supported. The models controlled for the community level of very severe abuse. Hence, the findings suggest that even when communities have high levels of physical abuse of children, those communities which are also high in Abiriwatia childcare values may be somewhat protected, compared to those that do not. Within Ghana, encouraging positive and protective aspects of traditional Ghanaian values and working to reinstate respect for these values may have positive outcomes for children. Moreover, the negative association found for Abiriwatia childcare values suggests the need for further study to unpack the community mechanisms by which these values are implemented and to understand more deeply whether the values encourage good parenting or community practices to intervene when severity of physical discipline exceeds community thresholds for decency. Parsons (1937) argued that while commitment to values may often be ubiquitous, it is how values act to regulate behaviors that determines their concrete effects. Therefore, it may be the case that commitment to Abiriwatia childcare values among caregivers in Ghana is unquestioned, but transformation of these commitments into regulation of parenting practices (to influence physical abuse) may be less common. As such, you may find high rates of physical abuse in Ghana (43% as of 2011, Akmatov, 2011), even where Abiriwatia childcare values are positively sanctioned. Our findings call for the need to strengthen both commitments to Abiriwatia values of childcare and enhance the regulatory mechanism that increases their impact on parenting and childcare. These findings also have implications for the wider world, as distinctly protective aspects of Abiriwatia community values could be encouraged and taught in other contexts around the globe. Such community values might be particularly protective for children growing up in poverty in individualistic cultures. Further research is needed to better understand the mechanisms by which protective Abiriwatia values relate to very severe physical abuse, and the means by which the values may be taught and transmitted. Given the results found for Abiriwatia for both neglect (Abdullah et al., 2023, forthcoming) and very severe abuse, we suggest that community interventions to reduce child maltreatment be developed with reference to Abiriwatia childcare values. Prevalence of physical abuse is extremely high in Ghana. However, many communities appear to have developed adaptations in response to these high levels of abuse to mitigate the violence and prevent serious injury and death. Researchers should not dwell exclusively on the bad news, but also place a strong focus on what Ghanaian communities have to teach the rest of the world about child protection.

Based on the previous research like that of Hiraoka et al. (2016), hypothesis 2 posited a positive relationship between borderline personality features and very severe physical abuse. People who have high levels of borderline personality features may suffer from rejection sensitivity (Downey & Feldman, 1996), experiencing labile emotions and the rapid onset of rage when they (mis)perceive rejection (Berenson et al., 2011). Our findings suggest that female caregivers with high levels of BPD features report higher levels of very severe abuse of children in Ghana, supporting hypothesis 2. Perceived rejection by children may be a risk factor for very severe abuse particularly when female caregivers perceive the child as being an important source of comfort, meaning, or friendship in their lives. Moreover, the findings suggest that the relationship between BPD features and very severe physical abuse is nonlinear. Diagnostics and simpler models found a significant relationship between BPD features squared and very severe abuse (*B* = 0.008, *p* < .05). Although the BPD features squared coefficient reverted to only marginal significance when all functional forms of focal child’s age were controlled, the significance of the squared term resurfaced in the interaction model, where it was highly significant (*B* = 0.07, *p* = .001).

Effectively, the model suggests the possibility of exponential growth in risk for very severe abuse at very high levels of BPD features. The results suggest that high BPD features are a risk factor for abuse in Ghana, not just in Western contexts. Because the forms of abuse (beating up, knocking down, choking, burning, and using or threatening to use a knife or gun) studied are potentially life threatening, it is vital to develop methods for recognizing high risk families and regularly following up with them. With training, communities could play an informal role in helping to monitor the well-being of kids at high risk for severe physical abuse.

Finally, the study hypothesized a negative interaction between community-level Abiriwatia childcare values and BPD features (Hypothesis 3). Although the linear interaction term was not significant, the interaction between Abiriwatia childcare values and BPD features squared was negative and highly significant (*B* = −0.001, *p* = .004), supporting hypothesis 3. Rather than a consistent interaction across all levels of BPD features, the result suggests that Abiriwatia childcare values at the community level may mitigate against exponential growth in risk at the highest levels of BPD features. The finding is encouraging because BPD is generally poorly understood, and yet communities high in Abiriwatia values may reduce the impact of the most severe BDP features. Further research to examine potential mechanisms for Abiriwatia effects could be used to underpin community education efforts. It is possible, for example, that communities with high levels of Abiriwatia values are more likely to become aware of mothers with high BPD features risk for severe abuse. Such communities may then intervene to mitigate that risk. Such efforts could educate communities about BPD to build empathy for both sufferers and their children, as well as to help community members understand the perspectives that people with BPD hold and what interventions they may most positively respond to. The findings suggest a little community efficacy against the BPD features—very severe abuse relationship, but existing protective effects might be substantially enhanced with community training and better understanding about BPD and other mental health issues. Moreover, the informal social control component of collective efficacy at the community level also had a strong and significant negative relationship with very severe abuse. This requires further research as high levels of informal social control may be one mechanism by which Abiriwatia childcare values translate into informal child protection practice in communities (Abdullah et al., 2023). It is also interesting that, controlling for the number of children in the household, the number of people in the household had a significant negative relationship with very severe abuse. This is consistent with Abdullah and Emery (2023) and Emery et al. (2020), who argued that more adults increased the possibility of effective social control to prevent or mitigate abuse and neglect.

The findings of this study for the first time examine Abiriwatia values and BPD features as they relate to acts of very severe physical abuse in a large nationally representative sample. The results are encouraging for two reasons: first, because they suggest that traditional Ghanaian values at the community level may be protective against severe physical abuse in and of themselves. Second, Ghanaian values at the community level may also mitigate the impact of individual-level risk factors for severe physical abuse. Such communities have much to teach the rest of the world. At the same time, complacency is not at all warranted. Too many children in Ghana confront severe and potentially life-threatening abuse on an annual basis. This is particularly true of rural communities where children are younger and very severe physical abuse is significantly more common.

### Limitations

This study has several limitations. The study findings cannot be generalized beyond the Ghanaian context. Also, evidence from the hypotheses examined do not indicate causation. Hence, they should be interpreted with caution. Because caregivers self-reported their perpetration of very severe physical abuse and self-endorsed the MSI-BPD checklist, there could be self-report bias. However, we hope to have minimized this risk through the secrecy measure, which is a measure of social desirability of admitting to perpetration of child maltreatment. The study is limited to only female caregivers in Ghana. Studies that involve children and male caregivers would be useful to extend the findings. The concern of nonlinearity in our models renders the findings conservative. Even though we have thoroughly vetted the concern of nonlinearity in our models, through diagnostics and regressing the squared, cube, and fourth power terms of the variables with greater concern for nonlinearity, studies using experimental techniques could help to further substantiate our findings. Studies from other contexts, especially African countries, are needed to replicate the findings to better shape community practices that protect children. Qualitative studies should be carried out to unravel the mechanisms that underlie the relationship between having high levels of BPD features and Abiriwatia values on childcare. Such studies should explore how people with BPD features perceive reality, and ways in which their notions about reality impact their responses to community norms on parenting practices. Research on positive parenting practices that are regulated by Abiriwatia values and how these practices negatively sanction very severe physical abuse would be useful to move the field forward.

### Conclusion

This study is important as the first empirical test of the relationship between the value of Abiriwatia and physical abuse severity in Ghana and, to the best of our knowledge, the first study in Ghana to examine the association between very severe physical abuse and female caregiver’s borderline features. The findings suggest that almost three out of every four children in Ghana have experienced some form of physical abuse in the past year, and the risk of severe physical abuse of children increases when their caregivers have higher BPD features. On a positive note, living in communities that are high in the Abiriwatia values of community childcare and respect for community authority could mitigate against the risk of physical abuse severity. Also, the Abiriwatia value of community childcare could mitigate the risk of high levels of BPD features, leading to fewer severe physical abuse cases. Altogether, the findings indicate the importance of Abiriwatia values and the need to increase understanding of BPD features and the risk they pose to children. Community programs that support both the institutionalization of Abiriwatia values and their internalization within Ghanaian people are important as part of community-based protective measures against severe physical abuse. Clinicians in Ghana should increase community sensitization through media outreach programs on BPD features and their consequences for children.

## Notes

1. Influence: “In the case of influence, alter refers to his experiences, his reasons, and his ‘potentiality or reasoned elaboration,’ which indicate a certain course of action to ego, who is expected to direct his intentions accordingly. . .It lacks a specialized supporting system and a symbiotic base and lives to a large extent on overdrawing truth” (Luhmann, 1976; p. 516).
